# Fish Poxviruses on the Rise: Prospects for Aquatic Health

**DOI:** 10.1111/jfd.70200

**Published:** 2026-05-08

**Authors:** Mikolaj Adamek, Marek Matras, Oluwaseun Christianah Ojelade, Motohiko Sano, Mona C. Gjessing, Tomas Korytar, Alberto Falco, Krzysztof Rakus, Verena Jung‐Schroers, Andor Doszpoly

**Affiliations:** ^1^ Fish Disease Research Unit, Institute for Parasitology University of Veterinary Medicine Hannover Hannover Germany; ^2^ Department of Parasitology and Invasive Diseases, Bee Diseases and Aquatic Animal Diseases National Veterinary Research Institute Pulawy Poland; ^3^ Department of Aquaculture and Fisheries Management Federal University of Agriculture, Abeokuta (FUNAAB) Abeokuta Ogun State Nigeria; ^4^ Department of Marine Biosciences Tokyo University of Marine Science and Technology Tokyo Japan; ^5^ Institute for Aquaculture Biotechnology (IAB) Tokyo University of Marine Science and Technology Tokyo Japan; ^6^ Norwegian Veterinary Institute Ås Norway; ^7^ Laboratory of Fish Immunology, Institute of Parasitology Biology Centre, Czech Academy of Sciences České Budějovice Czechia; ^8^ Fish Pathology Group Institute of Aquaculture ‘Torre de la Sal’ (IATS‐CSIC) Castellon Spain; ^9^ Department of Evolutionary Immunology, Institute of Zoology and Biomedical Research, Faculty of Biology Jagiellonian University Krakow Poland; ^10^ HUN‐REN Veterinary Medical Research Institute Budapest Hungary

## Abstract

Fish poxviruses are increasingly recognised as emerging pathogens of fish and should be considered in cases of unexplained gill or skin pathology. Carp edema virus (CEV), the causative agent of koi sleepy disease in common carp and koi ( *Cyprinus carpio* ), represents the first known example. Since then, additional members of the poxviridae have been described, including *Plecoglossus altivelis* poxvirus (PaPV) in ayu (*Plecoglossus altivelis*), seahorse poxvirus (SHPV) in Cape seahorse ( *Hippocampus capensis* ), salmon gill poxvirus (SGPV) in Atlantic salmon ( *Salmo salar* ), cod gill poxvirus (CGPV) in cod ( *Gadus morhua* ), black bullhead poxvirus in black bullhead ( *Ameiurus melas* ) and Japanese seabream poxvirus (JSPV) in red seabream ( *Pagrus major* ). Most poxviruses share a tropism for epithelial tissues, causing gill hyperplasia, lamellar fusion and in the case of SHPV, dermatopathy. Clinical presentation is often complicated by secondary infections due to the immunomodulatory effects of poxviruses. Diagnostic progress is hampered by their failure to replicate in cell culture, inconsistent electron microscopy results and the lack of broad molecular screening tools. Fish health professionals should remain vigilant and include poxviruses in differential diagnoses for gill and skin disorders.

## 1 Emergence of Fish Poxviruses

Following several new detections in recent years, poxviruses represent a rapidly expanding group of newly identified viral pathogens in fish, in terms of both host range and species diversity. For many years, reports largely focused on carp edema virus (CEV) in common carp and koi ( *Cyprinus carpio* ) (Hedrick et al. 1997; Lewisch et al. 2015; Miyazaki et al. 2005; Murakami et al. 1976; Oyamatsu et al. 1997) and poxvirus‐like infections in ayu ( *Plecoglossus altivelis* ) (Wada et al. 2008). These were considered sporadic and limited to specific geographical areas. However, the situation has changed markedly over the past two decades (Table 1), beginning with the characterisation of salmon gill poxvirus (SGPV) in Atlantic salmon ( *Salmo salar* ) (Gjessing et al. 2018, 2017, 2015; Nylund et al. 2008) and emergence of CEV to a global pathogen (Jung‐Schroers et al. 2015; Matras et al. 2017; Way et al. 2017; Zhang et al. 2017). In the case of SGPV, until then, undescribed gill disease was detected in Norwegian farmed Atlantic salmon about 30 years ago. The disease repeatedly appeared in certain hatcheries and was characterised by extensive apoptosis of the gill epithelium and acute, high mortality. A poxvirus was also observed in apoptotic epithelial cells (Gjessing et al. 2015; Nylund et al. 2008) by transmission electron microscopy (TEM). In 2015, the genome of SGPV was sequenced, and PCR and immunohistochemistry were developed, enabling the linkage of histopathology to the causative agent. Furthermore, RNA scope in situ hybridization (Thoen et al. 2020) has also been developed as a powerful tool to further characterise salmon gill poxvirus disease (SGPVD). Following the initial detection of CEV in Japan in the 1970s, the development of the first PCR method and its subsequent detection in the USA in the 1990s, a significant breakthrough occurred when CEFAS described the detection of a ‘CEV‐like’ virus in the UK (later designated as CEV genogroup I). This refined PCR methodology led to the detection of CEV worldwide (Jung‐Schroers et al. 2015; Matras et al. 2017; Way et al. 2017; Zhang et al. 2017). This was followed by the identification of additional fish poxviruses in Cape seahorse ( *Hippocampus capensis* ) (Groff et al. 2017), common bully ( *Gobiomorphus cotidianus* ) (Perry et al. 2022), Atlantic cod ( *Gadus morhua* ) (Gjessing et al. 2025), black bullhead ( *Ameiurus melas* ) (Abonyi et al. 2025), red seabream ( *Pagrus major* ) (Ishibashi et al. 2025), Sailfin tang ( *Zebrasoma velifer* ), two‐lined monocle bream ( *Scolopsis bilineata* ) and an unidentified dragonet species (*Synchiropus* sp.) (Costa et al. 2025).

**TABLE 1:** Currently known fish poxviruses.

Virus name	Host species	Disease	Clinical signs	Year (first report)	References
Carp edema virus (CEV)	Common carp, koi (*Cyprinus carpio, Cyprinus rubrofuscus*)	Koi sleepy disease (KSD)	Lethargy, anorexia, respiratory distress, swollen gills with edema epithelial hyperplasia, fusion of gill lamellae, proliferative branchitis, excessive mucus, pale gills, sunken eyes, skin ulceration, high mortality.	1970s (Japan, formal description 1997)	(Oyamatsu et al. 1997; Murakami et al. 1976)
*Plecoglossus altivelis* poxvirus (PaPV)	Ayu ( *Plecoglossus altivelis* )	Atypical cellular gill disease (ACGD); ‘Boke disease’	Epithelial hyperplasia, fusion of gill lamellae, proliferative branchitis, atypical cell formation, respiratory distress, anorexia, high mortality in juveniles.	1980s–1990s (Japan)	(Ishikawa et al. 2022; Wada et al. 2011, 2008)
Seahorse poxvirus (SHPV)	Cape seahorse ( *Hippocampus capensis* )	Cutaneous hyperplastic vacuolar dermatopathy	Dermatopathy: cutaneous hyperplastic vacuolar lesions, epidermal cell cytopathic effect, necrosis, epidermal thickening, skin discoloration; associated with increased mortality in managed populations.	2002 (characterised 2017, South Africa)	(Groff et al. 2017)
Salmon gill poxvirus (SGPV)	Atlantic salmon ( *Salmo salar* )	Salmon gill poxvirus disease (SGPVD); associated with apoptosis of gill epithelial cells; Complex gill disease	Severe epithelial apoptosis in gills, lamellar fusion, hyperplasia, massive leukocyte infiltration, reduced respiratory efficiency, anorexia, lethargy, mortality particularly in fry and smolts; seasonal recurrence and chronicity.	2008 (Norway)	(Gjessing et al. 2018, 2017, 2015; Nylund et al. 2008)
Cod gill poxvirus (CGPV)	Atlantic cod ( *Gadus morhua* )	Gill‐associated, cardiorespiratory disease	Epithelial hyperplasia, lamellar fusion, respiratory distress, cardiorespiratory compromise, lethargy; linked to farmed cod mortality.	2025 (Norway)	(Gjessing et al. 2025)
Black bullhead poxvirus	Black bullhead ( *Ameiurus melas* )	Unknown	Discovered in a mass mortality event of juveniles, however unknown pathology due to bacterial and viral coinfections strongly influencing clinical signs.	2025 (Hungary)	(Abonyi et al. 2025)
Japanese seabream poxvirus (JSPV)	Red seabream ( *Pagrus major* )	Unknown	Associated with massive mortality of juveniles, skin darkening, lethargy, possible gill involvement.	2025 (Japan/Korea, marine aquaculture)	(Ishibashi et al. 2025)
*Zebrasoma velifer* poxvirus	Sailfin tang ( *Zebrasoma velifer* )	Unknown	Metagenomic detection from seemingly healthy adult fish.	2024 (Australia, Great Barrier Reef)	(Costa et al. 2025)
*Scolopsis bilineata* poxvirus	two‐lined monocle bream ( *Scolopsis bilineata* )	Unknown	Metagenomic detection from seemingly healthy adult fish.	2024 (Australia, Great Barrier Reef)	(Costa et al. 2025)
Toitoi poxvirus 1	Common bully ( *Gobiomorphus cotidianus* )	Unknown	Gill metatranscriptome from seemingly healthy fish.	2022 (New Zealand)	(Perry et al. 2022)

**FIGURE 1:**
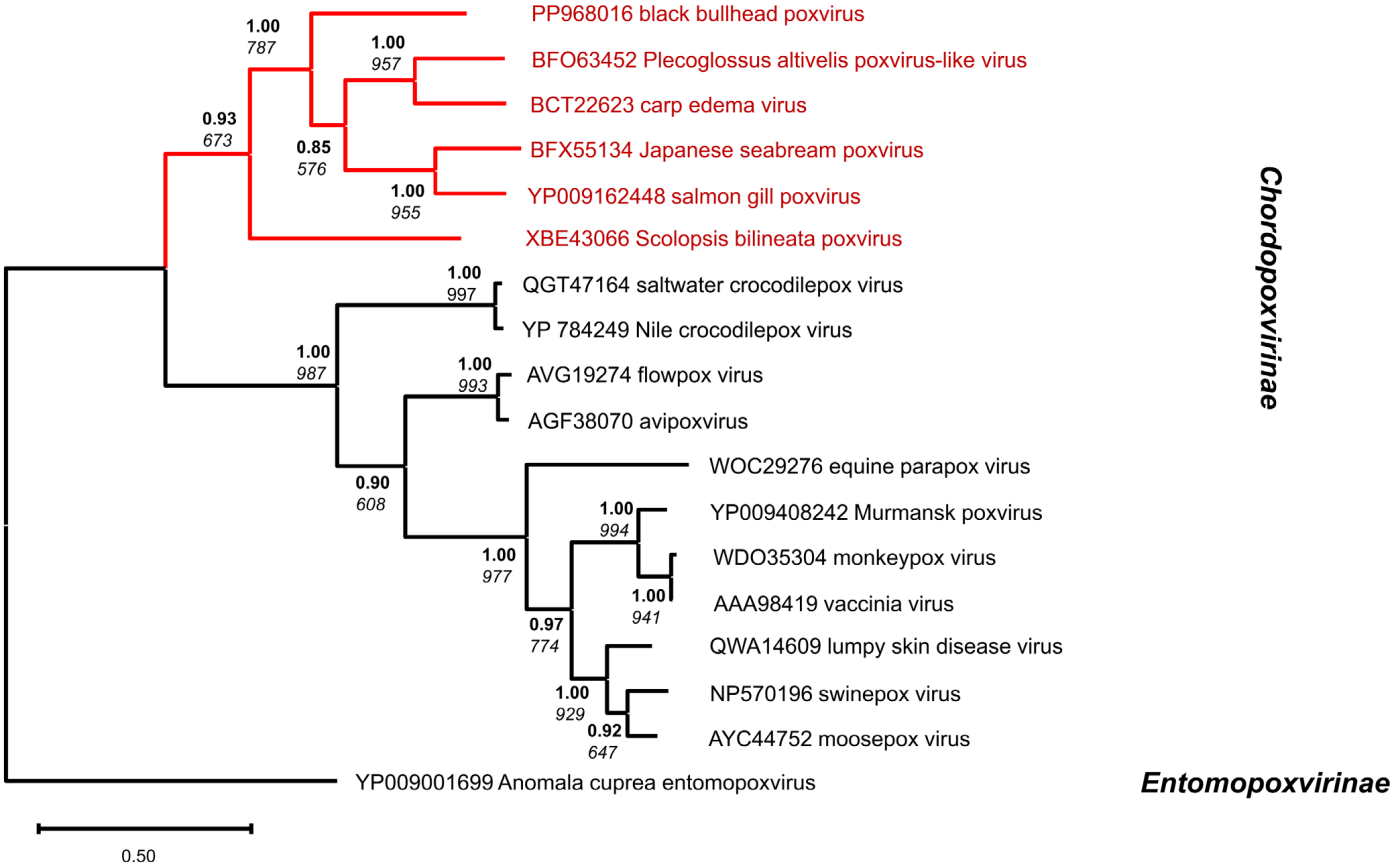
Fish poxviruses form a monophyletic clade within the family *Poxviridae*. The phylogenetic relations of poxviruses have been calculated with Bayesian analysis (1 million generations) and maximum‐likelihood (1000 replicates) method (CPR amino acid substitution model was applied for both methods) on the deduced amino acid sequences (177 aa) of the DNA polymerase gene. Detailed description of the alignment and the phylogenetic calculations have been published earlier (Abonyi et al. 2025). Anomala poxvirus from the subfamily *Entomopoxvirinae* was used as an outgroup. High statistical values (inference support for Bayesian analysis in bold and bootstrap values for ML in italics) confirm the topology of the tree. GenBank accession numbers of the viral DNA polymerases are given in front of the virus names.

Although genomes of some fish poxviruses have now been partially or fully sequenced, only one of them, the SGPV, has been assigned to an officially recognised virus species (*Salmonpoxvirus gillpox*) and genus (*Salmonpoxvirus*) by the International Committee on Taxonomy of Viruses, the rest of them remain unclassified (McInnes et al. 2023). Phylogenetic analyses based on conserved core genes consistently place fish poxviruses as a distinct and deeply branching lineage within the subfamily *Chordopoxvirinae* (Figure 1), separate from established genera for reptilian, avian and mammalian poxviruses such as *Crocodylidpoxvirus*, *Avipoxvirus*, *Orthopoxvirus*, *Capripoxvirus*, etc. This fish‐specific clade in the subfamily *Chordopoxvirinae* and clear separation of fish and tetrapod clades suggests that fish poxviruses are highly divergent from other known vertebrate poxviruses and have likely evolved from a single common ancestor (Costa et al. 2025; Perry et al. 2022). The organisation of fish poxvirus genomes is largely similar to that of other poxviruses, with conserved genes shared in the central region of the genome (Baba et al. 2025; Gjessing et al. 2015; Mekata et al. 2021). This reflects the application of molecular techniques, particularly PCR and next‐generation sequencing, and a growing awareness of the role of poxviruses in the pathology of fish species in aquaculture. Fish poxviruses should now be recognised as important agents of gill diseases and dermatopathies, which often predispose to secondary bacterial or parasitic infections and substantial production losses (Gjessing et al. 2020; MacNeill et al. 2025; Zawisza, Chadzinska, et al. 2024). The rapid pace of these discoveries highlights the urgent need to systematically re‐evaluate their evolutionary origins and their epidemiological impact on wild and farmed fish populations.

## 2 Disease Manifestation

Clinically, infections with the fish poxviruses manifest as a variety of signs that differ by host species, often presenting as pathology of the epithelium of gills and skin. In carp, CEV causes koi sleepy disease, characterised by profound lethargy, anorexia and oedema and dysfunction of the gills due to hyperplasia and occlusion of the gill lamellae, resulting in high mortality due to disorder of osmoregulation (Ono et al. 1986; Way et al. 2017). In salmon, SGPVD is associated with severe gill epithelial apoptosis, resulting in respiratory impairment and predisposition to secondary infections and sometimes complex gill disease (Gjessing et al. 2020, 2017). Furthermore, in some SGPVD cases, extensive erythrophagocytosis is seen (Benedicenti et al. 2025; Gjessing et al. 2020). Similarly, *Plecoglossus altivelis* poxvirus (PaPV) infections are associated with gill disease causing hypertrophy of gill epithelial cells, the formation of atypical cells containing basophilic inclusion bodies in the cytoplasm, lamellar fusion, aneurysms and epithelial necrosis (Ishikawa et al. 2022; Wada et al. 2011, 2008). In cases of CEV and PaPV co‐infection with *Flavobacterium branchiophilum* sometimes occur, which is also the causative agent of bacterial gill disease (Adamek et al. 2018; Ishikawa et al. 2022). Seahorse poxvirus (SHPV) however primarily produces cutaneous hyperplastic lesions in skin (Groff et al. 2017). A more recently discovered poxvirus in cod, named cod gill poxvirus (CGPV), has been associated with extensive pathology of gill epithelial cells and lamellar fusion with many affected cells staining positive for CGPV (Gjessing et al. 2025). Japanese seabream poxvirus (JSPV) has been linked to mortality, lethargy and darkening of the skin and the virus has been reliably detected in the gills (Ishibashi et al. 2025). Increased mortality in black bullhead has led to the detection of black bullhead poxvirus, but due to coinfections with bacteria (*Vibrio cholera* and *Plesiomonas shigelloides* ) and other viruses (*Ictavirus ictaluridallo2*), its clinical signs and pathological profile remains poorly defined (Abonyi et al. 2025). *Zebrasoma velifer* poxvirus, *Scolopsis bilineata* and *Synchiropus* sp. poxvirus have been reported from adult specimens showing no clinical signs and were detected during a metagenomic survey for microbial species in the Great Barrier Reef in Australia (Costa et al. 2025). Also, the Toitoi poxvirus 1 has an unknown disease status, as it was detected in the gill metatranscriptome of seemingly healthy common bully (Perry et al. 2022). Taken together, most of these infections highlight the significance of poxviruses as emerging drivers of gill and skin pathology in various species of farmed and wild fish. They frequently act in concert with environmental stressors and secondary pathogens (Adamek et al. 2022; Gjessing et al. 2017; Zhou et al. 2025).

## 3 Pathogen‐Host‐Environment Unknowns

Despite recent advances, there are still substantial knowledge gaps in our understanding of the biology of fish poxviruses. Most have been identified through limited molecular signatures of the viruses, with only a handful characterised by full genomes or even fewer successful laboratory infection models (Adamek et al. 2017; Ishikawa et al. 2022; Thoen et al. 2020). Consequently, their replication biology and host–virus interactions remain poorly understood (Benedicenti et al. 2025; Ouyang et al. 2023; Zawisza, Rebl, et al. 2024). The epidemiology and transmission dynamics of these viruses are largely unknown, including their potential reservoirs and the role of subclinically infected carriers (Adamek et al. 2022; Ishibashi et al. 2025; Matras et al. 2019). Furthermore, the immune responses and pathophysiological mechanisms underlying the disease presentations are not well understood, which complicates diagnosis, control and vaccine development (Adamek et al. 2021; Machat et al. 2024; Pikula et al. 2021). Addressing these gaps is essential for fully assessing the threat posed by this rapidly growing group of viruses to aquaculture and wild fish populations.

Fish poxviruses exhibit several unique biological and pathological features that distinguish them from other viral pathogens in fish. Like their terrestrial counterparts (Hernaez and Alcamí 2024), they encode a wide array of immunomodulatory proteins that interfere with host innate defences, including inhibitors of interferon signalling and molecules predicted to modulate cytokine responses (Gjessing et al. 2020; Ouyang et al. 2023). This capacity for immune evasion likely underpins the severe and often protracted pathology observed in infected fish, where massive epithelial hyperplasia in the gills or skin results in severe impairment of gill function or dermatopathies (Groff et al. 2017; Zawisza et al. 2025; Zawisza, Rebl, et al. 2024), but also abnormalities in other organs (Benedicenti et al. 2025). Interestingly, terrestrial poxviruses generally inhibit apoptosis (Hernaez and Alcamí 2024), in contrast to fish poxviruses which induce apoptosis of epithelial cells (Gjessing et al. 2020; Ouyang et al. 2023). This divergence likely reflects lineage‐specific selection shaping regulatory gene repertoires that underpin these opposing strategies.

Fish poxviruses appear to retain the general chordopoxvirus pattern of epithelial tropism (MacNeill et al. 2025), yet their epidemiology differs markedly due to the water milieu in which they spread. In fish, transmission is predominantly associated with direct horizontal spread through the water, especially via cohabitation and exposure of the gills and skin to viruses. The expression of the disease is strongly modulated by water temperature, husbandry and stress (Amundsen et al. 2021; Thoen et al. 2020; Zawisza, Rebl, et al. 2024). Water is a denser matrix than air and provides viruses with the ability to spread relatively easily (Gibson 2014); however, the other microorganisms suspended in water produce a plethora of enzymes that can significantly influence the infectivity of enveloped viruses over time (Oidtmann et al. 2018; Ullrich et al. 2021). By comparison, tetrapod chordopoxviruses show a broader mix of transmission methods, including direct contact, lesion‐to‐lesion spread, respiratory/aerosol spread in some systems, fomites and vector‐assisted transmission (Haller et al. 2014; Milton 2012). Orthopoxvirus transmission is often dominated by contact with lesions, scabs or contaminated materials, whereas avipoxviruses are well known for mechanical transmission by mosquitoes and other biting arthropods (Haller et al. 2014). Capripoxviruses and the lumpy skin disease virus can also involve blood‐feeding arthropods as important mechanical vectors, although direct or indirect contact may also contribute (Aleksandr et al. 2020; Namazi and Khodakaram Tafti 2021). The role of vectors is therefore one of the clearest contrasting features. In tetrapods, vector involvement is well established and forms part of standard epidemiological thinking with regard to avipoxvirus and lumpy skin disease. Thus, the ecological context is the main differentiator between poxviruses in fish and in tetrapods.

Another remarkable trait of fish poxviruses is their apparent long‐term low‐level and/or asymptomatic persistence in the host. The lines of evidence supporting these two possible ways of persistence include: (i) the subclinical long‐term carriage with asymptomatic shedding months after KSD recovery (Gorgoglione et al. 2022), (ii) the high prevalence of CEV‐positive batches among asymptomatic imported koi fish (Adamek et al. 2016; Montacq et al. 2025), (iii) the synchronous disease outbreaks occurring after stressful events, such as in black bullhead that were fished and restocked in research facilities, but not in uncaught fish (Abonyi et al. 2025). In addition, their mucosal tissue tropism and ability to induce host immunosuppression, further complicates their control. Together, these features highlight fish poxviruses as emerging and unusually complex threats to fish health and aquaculture biosecurity.

## 4 Comparison to Other DNA Virus Groups Pathogenic for Fish

Compared to other major fish viral pathogens with large DNA genomes, such as alloherpesviruses (e.g., *Cyvirus cyprinidallo3* formerly known as cyprinid herpesvirus 3—CyHV‐3) and iridoviruses (e.g., megalocytiviruses, infection spleen and kidney necrosis virus—ISKNV), fish poxviruses display a unique combination of traits that magnifies their impact. Like alloherpesviruses, they most probably can persist in hosts for long periods of time (Quijano Cardé and Soto 2024). However, unlike the relatively well‐characterised latency of for example CyHV‐3, the persistence of CEV or SGPV is not related to latency, which is not compatible with the biology of poxviruses, due to entirely cytoplasmic replication cycle. It is more often associated with subclinical carriage and recurrent outbreaks after stressful events (similar to alloherpesviruses) (Abonyi et al. 2025), which complicates detection and management. Compared to both iridoviruses and alloherpesviruses, which cause acute systemic infections with broad tissue tropism with high mortality (Hanson et al. 2011; Rakus et al. 2013; Zhu et al. 2021), poxviruses often induce tissue‐specific pathology, notably gill epithelial apoptosis, hyperplasia and dysfunction, that can cause significant morbidity and mortality by inducing acute and systemic disease, even in the absence of overt systemic viral replication. Most strikingly, poxviruses seem to encode a broader range of immunomodulatory genes than herpes‐ or iridoviruses (Aoki et al. 2007; Gjessing et al. 2015; Kushala et al. 2025; Mekata et al. 2021). This reflects their long co‐evolution with their fish hosts and enables them to suppress adaptive immune responses (Adamek et al. 2021), modulate apoptosis (Gjessing et al. 2015) and most likely also exploit host stress physiology (Thoen et al. 2020; Zawisza, Rebl, et al. 2024). These feathers make them more deadly and should make them increasingly recognised as a major threat to the health and sustainability of aquaculture.

## 5 Diagnostic Challenges and Recommendations

The diagnosis of fish poxviruses remains particularly challenging and requires us to reconsider current approaches. Unlike many other viral pathogens of fish, poxviruses have so far proven difficult to cultivate in cell cultures with only a very distinct Canadian isolate of SGPV somehow culturable (LeBlanc et al. 2019), which eliminates a key aspect of virological diagnostics and experimental work (Felten et al. 2022). Their large, complex virions are also sometimes surprisingly difficult to reliably detect by electron microscopy, particularly in mixed gill pathologies where secondary infections mask viral cytopathic changes. Molecular screening is further complicated by the lack of broadly reactive universal (degenerated) primers. Significant genetic diversity exists across known fish poxviruses, and an insufficient number of fully characterised genomes are available to establish robust, standardised pan‐poxvirus diagnostic protocols. Likely for this reason, they were not included in a pan‐pox qPCR assay developed to target entomopoxviruses and chordopoxviruses (Luciani et al. 2021). In this context, a recent publication describes a more inclusive primer design capable of detecting all JSPV genogroups as a good first step to improved diagnostics (Ishibashi et al. 2025). Further efforts to characterise new fish poxvirus genomes will be essential for the future development of pan‐piscine, or even broader, pan‐poxvirus assays, as well as for the completion of more comprehensive and refined taxonomic studies that will help to elucidate poxvirus evolution and the phylogenetic placement of fish poxviruses.

**FIGURE 2:**
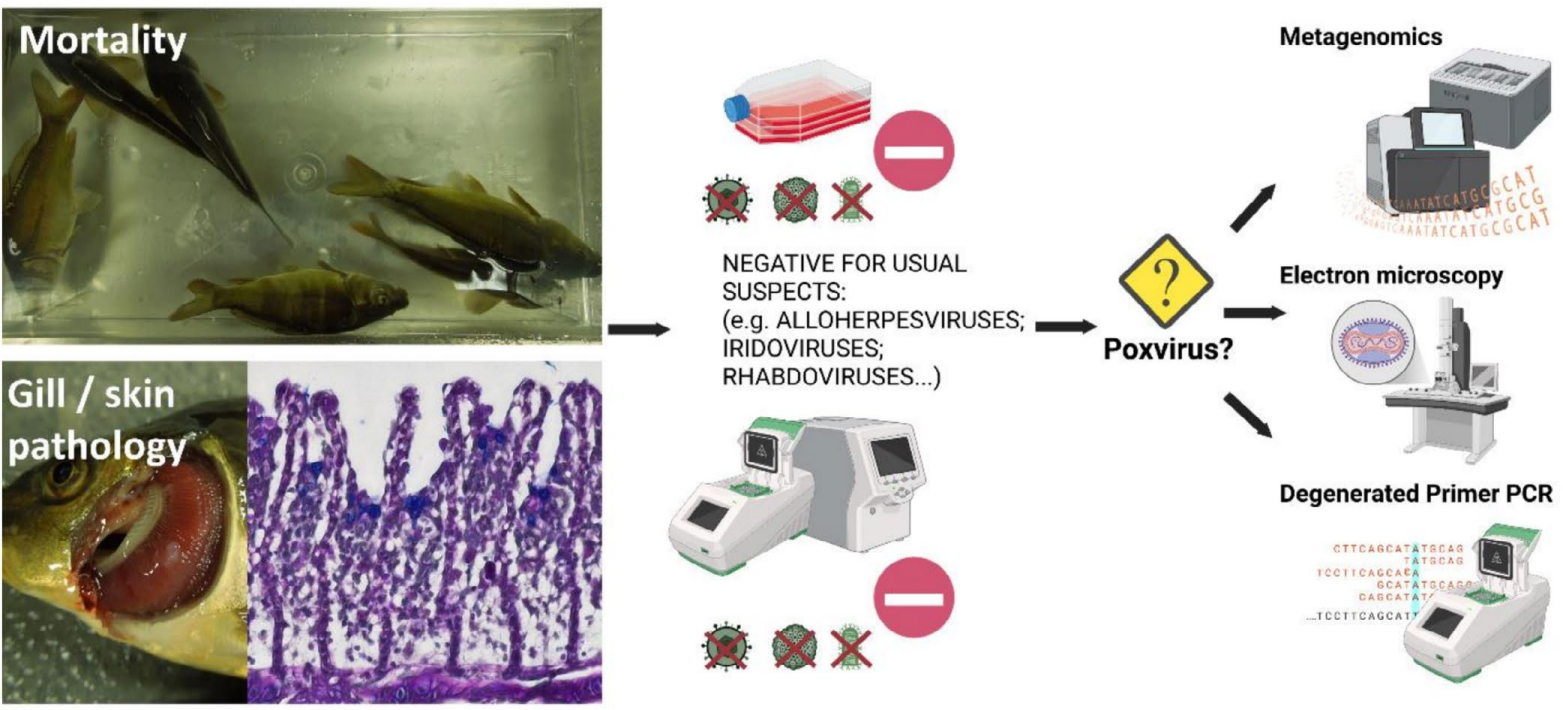
Diagnostic procedure in case unusual viral aetiology suspicion. Created in https://BioRender.com.

Another major challenge lies in the strong immunomodulatory capacity of these viruses, which predisposes the host to secondary bacterial, parasitic or fungal coinfections, which often dominate the histopathological picture and obscure the underlying viral aetiology (Adamek et al. 2019). Here, studies describing microbiome or pathobiome of poxvirus infected fish can lead to identification of bacterial species which are often detected in poxvirus infected fish and therefore represent strong indicators of poxvirus infection like *F. branchiophilum* in case of CEV (Adamek et al. 2018; Zawisza et al. 2026; Zhou et al. 2025). Evaluating immune responses during unexplained mortalities could also be helpful, as poxviruses appear to strongly induce an antiviral response (Zawisza, Rebl, et al. 2024). Also, development and accessibility to pathomics (Bülow et al. 2023), integrating ‘‐omics’ like transcriptomics (Gjessing et al. 2020; Ouyang et al. 2023), proteomics (Adamek, Majewska, et al. 2026; Machat et al. 2024), metabolomics (Adamek et al. 2021; Pikula et al. 2021) into the investigation of pathological causes, may be useful for detecting poxviral infections in fish, although these approaches may only be available in large laboratories with a broader focus on infectiology and are still too costly to apply to each case. For routine diagnostics, recurring epithelial alterations in the gills, including apoptosis, hyperplasia and lamellar fusion, should be considered a hallmark of poxvirus infections in fish and should remain the primary focus of diagnostic investigations and case definition refinement, especially in the absence of other ‘usual suspects’, i.e., common pathogens that cause gill pathology. These samples should be considered candidates for further investigation using more thorough electron microscopy, degenerate primer PCRs, or metagenomics (Figure 2).

## 6 Further Mitigation Strategies

In addition to reliable diagnostics, there are two fundamental strategies for mitigating the impact of poxviruses: breeding for host resistance and effective vaccination. Research on CEV indicates that resistance levels vary significantly between carp strains, and these differences are virus genogroup dependent (Adamek et al. 2017). However, implementing resistant fish strains in aquaculture is not straightforward, and this approach is not applicable to wild fish populations. The strong immunity developed in tetrapods in response to infection, and the impressive success of mammalian vaccination against poxviruses, provide a solid basis for fish poxvirus vaccines (Sánchez‐Sampedro et al. 2015). Furthermore, the conservation of certain core antigens across all poxviruses provides initial candidate targets for vaccines (Gjessing et al. 2015; Pacchioni et al. 2013; Papukashvili et al. 2022). Recently, DNA vaccines using plasmids encoding viral protein genes ORF227 of PaPV and ORF174 of CEV, homologues of the vaccinia virus core protein D13L, which are conserved within the *Chordopoxvirinae*, have been reported to exhibit preventive efficacy against PaPV and CEV (Baba et al. 2025; Ogawa et al. 2026). While further progress in vaccine development is anticipated, these vaccines may also serve as useful tools to investigate the immune mechanisms underlying protection against fish poxvirus infections that manifest as lesions in external mucosa tissues, such as the gill epithelium, in contrast to systemic infectious diseases in fish (Adamek, Zawisza, et al. 2026).

## 7 Conclusion—More Fish Poxviruses Ahead

Looking ahead, it is highly likely that additional poxviruses associated with fish diseases will be detected as surveillance intensifies and application of sequencing technologies advance further. As the saying goes, ‘each fish species is a host to its own unique alloherpesvirus, which just needs to be detected’, similarly the repeated discovery of novel fish poxviruses in phylogenetically and ecologically diverse fish hosts suggests that the currently known fish poxviruses catalogue represents only a fraction of their true diversity. Many cases of undifferentiated gill pathology or unexplained mortality events in aquaculture may in fact involve unrecognised poxvirus infections and should be investigated further as suggested here (Figure 2). Furthermore, the expansion of aquaculture and global trade in ornamental and food fish are expected to facilitate the emergence and dissemination of new poxviruses (Zawisza, Chadzinska, et al. 2024). Therefore, future research will need to integrate metagenomics, host–pathogen interaction studies (e.g., monitoring immune responses as a molecular clue for detection of pathogens) and improved diagnostic tools in order to better define the prevalence, pathogenic potential and risk to aquaculture sustainability of these viruses.

## Author Contributions

**Mikolaj Adamek:** conceptualization, visualisation, investigation, data curation, supervision, resources, writing – original draft, writing – review and editing, funding acquisition, project administration. **Marek Matras:** writing – original draft, writing – review and editing, data curation, investigation, conceptualization. **Oluwaseun Christianah Ojelade:** conceptualization, writing – original draft, writing – review and editing. **Motohiko Sano:** conceptualization, writing – review and editing, writing – original draft. **Mona C. Gjessing:** conceptualization, writing – original draft, writing – review and editing. **Tomas Korytar:** conceptualization, writing – original draft, writing – review and editing. **Alberto Falco:** conceptualization, writing – original draft, writing – review and editing. **Krzysztof Rakus:** conceptualization, writing – original draft, writing – review and editing. **Verena Jung‐Schroers:** conceptualization, writing – original draft, writing – review and editing, data curation. **Andor Doszpoly:** conceptualization, methodology, writing – original draft, writing – review and editing, data curation, visualisation.

## Funding

This study was supported by the joint project funded by the German Research Foundation (Deutsche Forschungsgemeinschaft, DFG, grant number 426513195, awarded to M.A.) and National Science Centre of Poland (grant number UMO‐2018/31/F/NZ6/02311, awarded to K.R.) under the Beethoven Life 1 project framework. M.A. was also funded by the European Union's Horizon Europe Research and Innovation Programme under grant agreement 101084204 Cure4Aqua. A.F. acknowledges the financial support from the Spanish Ministry of Science, Innovation and Universities and the State Research Agency (MICIU/AEI, https://doi.org/10.13039/501100011033) and the European Union NextGeneration EU/PRTR through the CNS2023 grant (project: AMPDANCE; ref.: CNS2023‐145412); as well as from the Conselleria de Educación, Cultura, Universidades y Empleo of the Generalitat Valenciana through the 2024 GVA AICO grant (project: TOMACUA; ref.: CIAICO/2024/281).

## Conflicts of Interest

The authors declare no conflicts of interest.

## Data Availability

The authors have nothing to report.
